# Mining physical protein-protein interactions from the literature

**DOI:** 10.1186/gb-2008-9-s2-s12

**Published:** 2008-09-01

**Authors:** Minlie Huang, Shilin Ding, Hongning Wang, Xiaoyan Zhu

**Affiliations:** 1 State Key Laboratory of Intelligent Technology and Systems, Tsinghua National Laboratory for Information Science and Technology, Department of Computer Science and Technology, Tsinghua Park No. 1, Tsinghua University, Beijing 100084, China

## Abstract

**Background:**

Deciphering physical protein-protein interactions is fundamental to elucidating both the functions of proteins and biological processes. The development of high-throughput experimental technologies such as the yeast two-hybrid screening has produced an explosion in data relating to interactions. Since manual curation is intensive in terms of time and cost, there is an urgent need for text-mining tools to facilitate the extraction of such information. The BioCreative (Critical Assessment of Information Extraction systems in Biology) challenge evaluation provided common standards and shared evaluation criteria to enable comparisons among different approaches.

**Results:**

During the benchmark evaluation of BioCreative 2006, all of our results ranked in the top three places. In the task of filtering articles irrelevant to physical protein interactions, our method contributes a precision of 75.07%, a recall of 81.07%, and an AUC (area under the receiver operating characteristic curve) of 0.847. In the task of identifying protein mentions and normalizing mentions to molecule identifiers, our method is competitive among runs submitted, with a precision of 34.83%, a recall of 24.10%, and an F_1 _score of28.5%. In extracting protein interaction pairs, our profile-based method was competitive on the SwissProt-only subset (precision = 36.95%, recall = 32.68%, and F_1 _score = 30.40%) and on the entire dataset (30.96%, 29.35%, and26.20%, respectively). From the biologist's point of view, however, these findings are far from satisfactory. The error analysis presented in this report provides insight into how performance could be improved: three-quarters of false negatives were due to protein normalization problems (532/698), and about one-quarter were due to problems with correctly extracting interactions for this system.

**Conclusion:**

We present a text-mining framework to extract physical protein-protein interactions from the literature. Three key issues are addressed, namely filtering irrelevant articles, identifying protein names and normalizing them to molecule identifiers, and extracting protein-protein interactions. Our system is among the top three performers in the benchmark evaluation of BioCreative 2006. The tool will be helpful for manual interaction curation and can greatly facilitate the process of extracting protein-protein interactions.

## Background

An important step in functional systems biology is to elucidate the relationships between biomolecules. Interactions between proteins define biological pathways, and knowledge of the processes in which proteins are involved is essential to gaining a fundamental understanding of the cellular machinery. The study of protein interactions is a pressing biological imperative, and thus characterizing protein interaction partners is crucial to our understanding of both the function of individual proteins and the organization of entire biological processes [1,2].

More and more interaction data are being published in the literature as a result of the development of high-throughput experimental technologies, such as the yeast two-hybrid screening and affinity purification coupled with mass spectroscopy. These experimental techniques make it possible to study protein interactions on a much larger scale, although they suffer at times from poor resolution. To provide reliable protein interaction data for biologists, interaction databases such as Molecular Interactions Database (MINT) [3] and IntAct [4] manually detect and curate protein interactions from different information sources. However, it is becoming difficult for database curators to keep up with the rapidly expanding literature and the increasing number of newly discovered proteins.

In addition to the rate at which interaction data are being produced, there are other challenges for manual interaction curation. Experimental methods are not equally reliable, and when they extract protein interactions curators must place emphasis on thorough description of the experimental evidence. Furthermore, many authors continue to use ambiguous gene or protein names in their reports, or they fail to provide the organism or tissue from which the genes or proteins originate. Difficulty in mapping gene and protein names to SwissProt/UniProt [5,6] identifiers increases the work of an annotator, who must gather more information from references, supplemental material, and so on. Finally, many types of interactions are scattered in the literature, although many are irrelevant to physical protein-protein interactions (for example, physical interaction [MI:0218] from the Molecular Interaction Ontology [7] is defined as interaction among molecules that can be direct). Genetic interactions (MI:0208: functional relationship among genes revealed by the phenotype of cells carrying combined mutations of those genes) are considered to be distinct from physical interactions between proteins, and they are not currently curated. Similarly, neither drug-drug interactions nor interactions between protein complexes and proteins are considered to be relevant in physical interaction curation.

Because of the accumulation of interaction data in the biomedical literature and the challenges that present for manual curation, there is an urgent need for text-mining tools to facilitate the extraction of such information. In particular, the extraction of physical protein-protein interactions, defined as the co-localization or direct interaction between protein molecules, is becoming extremely important because physical interactions are the most reliable data produced in high-throughput experiments. The development of effective text-mining tools could aid the mapping of proteins to SwissProt/UniProt identifiers, as well as the discovery of experimental evidence for interactions and the discrimination of physical interactions from other types of interactions. In comparison with previous studies on bio-text mining, BioCreative 2006 [8,9] addressed some of these difficulties, such as normalizing gene/protein mentions to molecule identifiers, discriminating physical interactions from other interactions, and gathering as much reliable experimental evidence as possible.

The protein-protein interaction (PPI) task of BioCreative 2006 is comprised of several subtasks: the interaction article subtask (IAS) to determine whether an article (abstract only) is relevant to some physical interactions; the interaction pair subtask (IPS), in which interacting protein pairs should be extracted from full-text articles; the interaction sentence subtask, in which participants are required to submit a list of summary sentences for each interaction; and the interaction method subtask, in which the experimental detection methods should be given for each interaction.

Here, we present the methods and results from our participation in the PPI task of BioCreative 2006 [10]. By using Kullback Leibler divergence, we study the quantitative divergence between the training data and the final test data in the IAS, indicating that this originates from not being able to provide an adequate set of irrelevant articles. We propose solutions to overcome this issue and, in addition to the term features, other features are studied to reduce the distribution divergence such as the string, entity, and template features. Information fusion from both the feature perspective and the classifier perspective is studied, and our results rank in first place in terms of accuracy and in second place in terms of area under the receiving operator characteristic curve (AUC) in the benchmark evaluation. With this improvement, the tool may be useful in practical interaction curation.

In addition, we propose a named entity recognition framework that utilizes the information on the organism in articles. We present a quantitative analysis of how the extraction of physical interactions is influenced by the errors caused by the named entity recognition module. We point out that the framework is extremely important because, in the interaction curation task, protein names must be normalized to molecule identifiers so that molecular properties such as sequence can easily be identified. Finally, a profile-based method is proposed for the IPS. The goal of the task is to extract the protein pairs that have experimentally verified evidence, which requires the curator to collect information from multiple sentences in the article. This goal is different from the general aim of PPI extraction systems, most of which extract PPIs at the sentence level. Inspired by experience in curation, we constructed a profile vector for each candidate interaction from the whole article. By integrating evidence from the whole article, a better prediction is achieved for robust interaction curation. Our results were ranked in first place in terms of F_1 _score on the SwissProt-only subset, and in second place on the entire dataset. However, these results clearly need improvement if they are to be useful for any real task.

## Results and discussion

### Article filtering for efficient interaction curation

Automatically filtering out articles that are irrelevant to interaction curation will be useful to database curators. According to the reports from database curation projects, it takes 2 to 3 hours to process a paper even for highly qualified curators [1]. The IAS of BioCreative 2006 specifically addressed this issue of how to assess an article filtering tool in order to facilitate this process [8]. The task is difficult because the relevance of some articles cannot be determined through reading their abstracts alone, and curators usually must obtain evidence from the full text. Moreover, articles describing genetic interactions are hard to separate from those with physical interactions.

In the training dataset, there are 3,536 articles relevant to physical interactions and 1,959 irrelevant ones, and the official test dataset has 375 relevant and 375 irrelevant articles. A serious problem in this task is that the performance with the training data is much better than that with the official test data (0.95 versus 0.80 in terms of F_1 _score), which was also observed by [11]. To analyze the problem, 750 articles (375 positive) were taken out of the training corpus at random and defined as the leave-out dataset. The top 50 features, whose significance was measured using the *χ*^2 ^test, were selected from the remaining training dataset. Based on these 50 features, three probability distributions were estimated from the leave-out dataset by using Equation 3 (see below), from the remaining training dataset, and from the official test dataset. We then calculated the average Kullback Leibler divergence (defined by Equation 4 [see below]) between two distributions to measure the divergence between distributions (Table 1), where the term features are unigrams/bigrams and the string features are strings with seven characters.

**Table 1:** The average Kullback Leibler divergence between the distributions of different datasets

Compared distributions	Term feature		String feature	
	
	Pr(x|c_+_)	Pr(x|c_-_)	Pr(x|c_+_)	Pr(x|c_-_)
Dist on the remaining training dataset versus Dist on the leave-out dataset	0.0216	0.0703	0.0029	0.0163
Dist on the remaining training dataset versus Dist on the official test dataset	0.0369	0.9926	0.0357	0.1887

The table shows the average Kullback Leibler divergence of three distributions estimated on the leave-out dataset, remaining training dataset, and the official test data. The Average Kullback Leibler divergence between distributions on different datasets. Dist, distribution.

**Figure 1:**
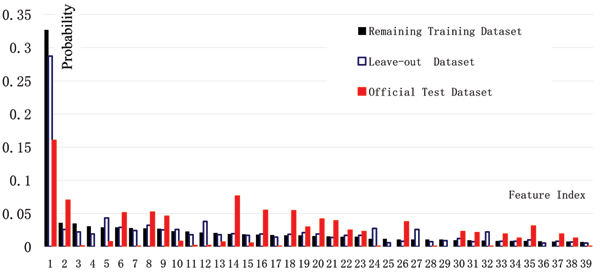
The probability of a feature x occurring in irrelevant articles. The figure shows the three distributions of the leave-out dataset, remaining training dataset, and official test dataset. The probability of a feature x occurring in irrelevant articles (Pr(x|c_-_)) in different datasets are shown (only 40 features are listed here).

For Pr(x|c_+_), the probability of a feature *x *occurring in the relevant articles, there was no remarkable difference between the term distributions estimated from the leave-out dataset, the remaining training dataset, or official test dataset. In other words, the three different datasets have almost the same term distribution. However, significant differences were observed for Pr(x|c_-_), the probability that a feature *x *appeared in the irrelevant articles, whose distributions are illustrated in Figure 1. There is much greater divergence between the distribution estimated from the official test set and that from the training dataset (0.992 versus 0.188). We hypothesize that the term distribution is different in the official test set, and that this may be the reason why the model did not hold well in the official test dataset. This was also verified by [11], in which much better performance was obtained when the training dataset and final test dataset were reversed. When the string is selected as a feature, the divergence diminished notably (0.992 versus 0.188), which might explain why the string feature proved even better than the term feature in these runs, as shown in Table 2.

**Table 2:** Article filtering performance with different features and classifiers

Model	Precision	Recall	F_1 _score	AUC
Mean	0.6642	0.7636	0.6868	0.7351
Standard deviation	0.0810	0.1926	0.1035	0.0741
Best reported in terms of AUC [8]	0.7080	0.8609	0.7770	0.8554
Our results in BioCreative 2006	0.7507	0.8107	0.7795	0.8471

Term (baseline)	0.7016	0.8213	0.7568	0.8037
String	0.7044	0.8960	0.7887	0.8416
Named entity (NE)	0.5815	0.9600	0.7243	0.7570
Template	0.7841	0.7653	0.7746	0.8239
String + NE	0.7360	0.8773	0.8005	0.8479
String + template	0.7416	0.8880	0.8082	0.8372
String + NE + template	0.7585	0.8373	0.7959	0.8507
String + term + NE + template	0.7432	0.8720	0.8025	0.8608

Naïve Bayes classifier	0.6321	0.8613	0.7291	0.7884
Multinomial classifier	0.6264	0.8720	0.7290	0.7770
Linear kernel SVM	0.7016	0.8213	0.7568	0.8037
*p*-spectrum kernel SVM (*p *= 7)	0.7352	0.8293	0.7794	0.8376

Integration of the above four classifiers (AdaBoost)	0.7995	0.8933	0.8438	0.8746

This table shows the experimental results from article filtering. AUC, area under the receiving operator characteristic curve; SVM, support vector machine.

We might conclude that the problem originates from the fact that the irrelevant articles are not sufficiently representative of the entire sample space. In the interaction curation task, irrelevant articles are more randomly distributed, where some articles describing genetic interactions are very similar to those dealing with physical interactions, some discuss other types of interactions (for example, drug-drug interactions), and some are completely different and can easily be filtered out. It is difficult to provide a good set of representative irrelevant articles, and so these irrelevant articles introduce more uncertainty and bias into the learning machines.

In an attempt to overcome the problem, we first took strings as features based on the above analysis. Furthermore, we propose a new scheme (defined by Equation 5 [see below]) to diminish the divergence between the training data and the test data. The new scheme takes into account the probability of a feature being observed in both relevant articles and in irrelevant ones, instead of simply using TF*IDF (term frequency × inverse document frequency). Alternatively, we tried to incorporate more high-level semantic features such as the named entity features and template features. The entities were recognized using ABNER (A Biomedical Named Entity Recognizer) [12], including the protein, DNA, RNA, cell line, and cell type. The TF was calculated for these entity features, and template features were exploited to represent the specific syntactic dependency between entities. In a third attempt, we integrated more information from different classifiers, and by fusing different classifiers the performance was boosted markedly.

We first studied how the features influence performance in terms of classification using a support vector machine (SVM) with a linear kernel as the classification model (Table 2). The string features easily defeated the term features (F score: 0.788 versus 0.756; AUC: 0.841 versus 0.803), and we speculated that this was because the string features are more powerful, thus eliminating the divergence between the training data and the test data (as mentioned above). Note that an attraction of the entity features is the very high recall obtained (0.96), indicating that almost all the original relevant articles have been selected out. This is very useful if the precision of the classification is to be improved by further processing. By integrating all of the features together, the AUC is further improved to 0.861.

Second, we studied how this issue is influenced by different classification models, each model analyzing the data from a different point of view. SVM learns to separate data by a decision hyperplane, whereas the naïve Bayes classifier and multinomial classifier estimate probability distributions and try to interpret data from the probability perspective. The linear kernel SVM requires the data to be represented as feature vectors, whereas the p-spectrum kernel SVM simply views an example as a string. The different description powers can be combined by AdaBoost [13] and the best performance approached the needs of practical usage, with a precision of 80% and a recall of 90%.

Readers should note that the results presented in Table 2 are significant at the 0.02 level because we performed *t*-test experiments to determine whether the observed improvements were statistically significant. More details are presented in our paper published elsewhere [14].

### Normalizing protein names to SwissProt identifiers

It is extremely useful to normalize protein names with molecule identifiers, which will largely ease the process of interaction curation. However, the task is challenging because inconsistent naming terminologies are used. It is common in reported research to cite just a few, nonstandard abbreviations, or to mention proteins without specifying species or organisms, or without specifying isoforms. This problem can be exemplified as follows.

1. Common terms, such as p53, are not easily normalized without any contextual information.

2. The same term is used to name different molecules from the same or related genes but different organisms. For example, PI3K may refer to different molecules in mouse (P42337), human (P42336), and cow (P32871), whose genes have the same term PIK3CA.3. The same term is used to name different isoforms of molecules. For example, in mouse PI3K refers to both Q8BTI9 (the *β *isoform of the protein) and O35904 (the *δ *isoform). There are two important steps in normalizing names to SwissProt identifiers. First, the terms of database entries must be curated to canonical forms, and the new terms used to detect the mention of proteins. Second, the ambiguities of multiple mapping of protein mentions to molecule identifiers should be removed by using organism and contextual information. The following rules are used to curate database entries.

1. The gene names/synonyms and gene product names/synonyms for the same entry are included.

2. Prefixes and suffixes that are not crucial for entity identification are removed. For example, the prefixes c, n, and a of PKC are removed where these prefixes mean conventional, novel, and atypical, respectively.

3. Terms with digits or Roman/Greek numbers are transformed into a unified format: alphabetical + white space + digits. This rule affects examples such as the following: IL-2 and IL2 become IL 2; and CNTFR alpha, CNTFR A, and CNTFR I become CNTFR 1.

4. Terms that are not in abbreviated forms are converted to lowercase.

About 230,000 normalized entries are produced from SwissProt database and, as mentioned previously, there are many ambiguous mappings to database identifiers, even with normalized terms. To solve these ambiguities, the nearest neighbor principle is used, based on the organism context. The presumption here is that each protein name belongs to a particular organism context. Organisms in each sentence are identified, and the organism context of a protein name is defined by organisms appearing in adjacent sentences. The organism of candidate proteins is determined by the nearest neighbor principle.

Our work on protein mention normalization is very simple and coarse, and there is still much room for improvement. There is a thorough study on how to create a dictionary by combining multiple gene/protein databases together [15]. A number of spelling variation rules were studied in that work, and the investigators pointed out that many rules appeared to have no effect and some appeared to have a detrimental effect on precision. These discoveries will be very useful in making improvements to our module in the future.

In the IPS of BioCreative 2006, 740 full-text articles are provided for training and 358 for testing. The articles were provided for evaluating the extraction of protein pairs, but there was no separate step to evaluate protein mention normalization. However, the organizers returned to participants the results related to normalization, which were evaluated in a different manner. Our results are shown in Table 3, in which the average results were based on 45 runs from 16 teams. These results were from the official evaluation but they were not published in the BioCreative workshop. Clearly, our results are better than the average results (ours > mean + dev). However, the results presented here are considerably poorer than the results obtained from the gene normalization (GN) task of BioCreative 2006. The best result for the GN task was a precision of 78.9%, a recall of 83.3%, and a F_1 _score of81.0%, which are significantly better than the results we present here. Readers should note the major differences between results in the PPI task and those in the GN task. First, the PPI task required participants to process both abstracts and full texts, whereas only abstracts were processed in the GN task. Second, the measurement in the two tasks was different. In the PPI task, the gold standard set was made up of only the protein molecules that have annotated interactions. Other correctly identified proteins without interaction annotation were not included in the gold standard set. In the GN task, the gold standard set included all the gene identifiers that should be normalized, and all of the submitted identifiers were evaluated.

**Table 3:** Comparative results for protein name normalization

		Precision	Recall	F_1 _score
Average	Mean	0.1495	0.2828	0.1707
	Standard deviation	0.0963	0.1294	0.0764
	Median	0.1337	0.2723	0.1683

Our results	Baseline	0.2223	0.1024	0.1402
	+ entry curation	0.2345	0.2648	0.2487
	+ organism context	0.3483	0.2410	0.2849

The table shows the comparative results when identifying and normalizing protein names.

### Physical interaction extraction

The module for interaction extraction will greatly facilitate the process of interaction curation for database curators. It is not always easy to identify a single sentence that clearly describes an interaction in a paper. However, most previous methods extract interactions at the sentence level [16-22], where each sentence is handled independently. Inspired by the fact that curators must gather sufficient evidence to decide whether an article claims that there is a physical interaction, we propose a profile-based method to extract physical interactions by integrating evidence from the whole document.

The results shown in Table 4 confirm that our method performs much better than others (ours > mean + 2 × dev). In the benchmark evaluation, our results rank first in terms of F_1 _score, second in terms of precision, and third in terms of recall on the SwissProt-only subset, whereas on the entire dataset our results were all in second place (F_1 _score, precision, and recall). Also, our method outperforms traditional template-based methods, considering that ONBIRES (Ontology-Based Biological Relation Extraction System) represents the state-of-the-art performance [22].

**Table 4:** Comparative results for interaction pair extraction

Compared models	Whole collection			SwissProt only article collection		
	
	Precision	Recall	F_1 _score	Precision	Recall	F_1 _score
Mean	0.1062	0.1858	0.1035	0.1160	0.2000	0.1127
Standard deviation	0.0945	0.1001	0.0761	0.1035	0.1062	0.0836
Median	0.0755	0.1961	0.0788	0.0808	0.2156	0.0842
Best reported in terms of F_1 _score [8]	0.3908	0.2970	0.2849	0.3893	0.3073	0.2885

Template-based method (threshold = 0.0)	0.1373	0.2905	0.1578	0.1566	0.3189	0.1784
Template-based method (threshold = 80.0)	0.2177	0.2651	0.2038	0.2434	0.2828	0.2247
Profile-based method	0.3096	0.2935	0.2623	0.3695	0.3268	0.3042

'Whole collection' means that all of the articles have been considered. 'SwissProt only article collection' include articles containing interaction pairs that can be normalized to SwissProt entries. The table shows the comparative results for the extraction of interaction pairs.

**Figure 2:**
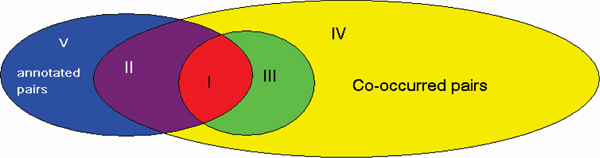
Errors of interaction pair extraction. The figure shows the distribution of errors in the interaction pair extraction. The blue ellipse contains 798 annotated pairs, the yellow ellipse 8,172 coincident pairs, and the green circle 339 extracted pairs. I, 100 true-positive samples; II, 166 coincident but false-negative samples; III, 239 false-positive samples; IV, 7,135 true-negative samples; V, 532 false-negative samples but never coincident.

There are reasons for the success of our profile-based method in this particular task. First, by integrating evidence from the whole article, the method is more robust when extracting physical interactions. For example, the sentence 'A interacts with B' will definitely be taken as a positive example by template-based methods, although it may describe a genetic interaction. In the profile-based method other evidence is required to make such a claim. Second, abundant features such as term features, entity features, template features, and position features are all integrated into the method. Here, we analyze the errors in detail in order to identify the problems hindering overall performance. There are 798 manually annotated interaction pairs in the 358 test articles, and although 339 protein pairs were extracted, only 100 of these are true positive pairs. There were 8,172 pairs that co-exist, and many of these include incorrectly recognized names (Figure 2). Among the 239 false-positive errors (area III), the first 50 errors were manually checked, and they fell into three categories.

1. Twenty-two errors were due to incorrectly normalized names. For example, in the sentence 'BAF60c interacts directly with PPAR gamma *in vitro*', the annotated interaction is Q6STE5 (BAF60c)-P37231 (PPAR gamma), and although we correctly extracted the interaction, the names were unfortunately normalized to Q6P9Z1-O19052.

2. Twelve errors were due to false-positive names in sentences where protein A and B physically interacted, and a false positive recognized protein C coupled to A or B.

3. Sixteen errors were due to the classifier, which included classifying nonphysical interaction pairs as exhibiting a physical interaction and other problems. This problem is partly due to the classification model and partly to the incompleteness of the training set, which does not provide evidence of samples that truly interacted.

Among the 698 false negative errors (areas II + V), the majority were caused by the identification and normalization of protein mentions (532 errors), whereas 166 were due to the interaction extraction model. In the 166 pairs that co-existed, we found that 37 were negatively classified because the sentences did not contain sufficient evidence. Examples of such sentences include 'A activates B' or 'Camptothecin-induced nuclear export of A does not require B'. This problem is also due to the fact that the evidence of physical interactions is not confined to a single sentence. The remaining 129 errors are believed to be caused by our classifier.

From these analysis, we conclude that the difficulty of protein name normalization leads to the majority of errors, producing about 64% (34/50) false-positive errors and 76.2% (532/698) false-negative errors. The second problem is that of incomplete annotation. Because the annotation only specifies the interacted protein ID in the article without the passages providing evidence or the location of these molecules, it makes the training process untraceable and the process of error analysis extremely difficult. Currently, a major limitation of our method is the requirement of protein coincidence within a sentence. This is not always the case in practical interaction curation, where curators often find evidence from contextual sentences, each of which may contain only one protein of the interacting pair. For example, the sentence 'the two proteins are co-purified together' may describe a physical interaction, even though both proteins are mentioned in the preceding sentences instead of in this sentence itself.

## Conclusion

In this report we discuss three key issues related to practical interaction curation. Specifically, we deal with filtering articles irrelevant to physical PPIs, identifying the mention of proteins and normalizing them to molecule identifiers, and extracting experimentally verified interactions. Different levels of features, including the string, term, named entity, and template features, are exploited to study the problem of distribution divergence between the training and test data. An AdaBoost-based information fusion technique is studied to integrate the various powers of description of different classifiers. Through these improvements, high-performance article filtering produces a system that may facilitate the process of interaction curation. Although the current state of protein name identification and normalization leaves much to be desired, our method utilizes the organism information to reduce ambiguity and, with further improvements, it will aid biologists. The profile-based interaction extraction method combines evidence from sentences in the whole document, thereby providing some improvement in the ability to predict physical interactions. In comparison with traditional methods that extract interactions at the sentence level, our method utilizes information from the whole article.

There are still many difficulties and challenges in extracting biologically meaningful knowledge, for example recognizing biological molecules with widely accepted identifiers and mining physical interactions with experimentally verified evidence. This report provides methods to help resolve these problems both from the perspective of the feature and that of the classifier.

## Materials and methods

**Figure 3:**
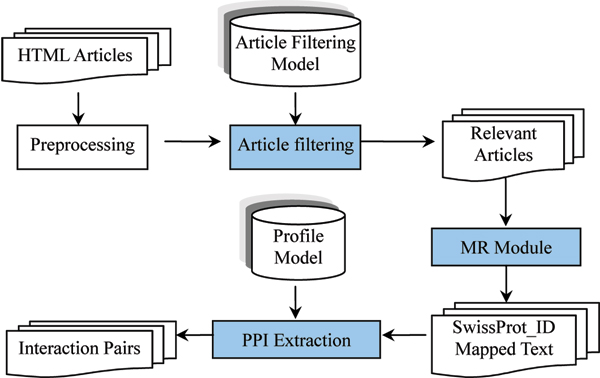
The system architecture of our method. Blue rectangles are the three main modules in our system. The figure shows the architecture of our system, and there are three main modules in the system that have been colored in blue. MR, molecule recognition; PPI, protein-protein interaction.

We first present the architecture of our method (shown in Figure 3), and then we describe the models and algorithms used in our method. There are three major modules in our framework: the first for filtering irrelevant articles; the second for identifying and normalizing protein mentions to SwissProt identifiers; and the third for extracting PPIs.

### Article filtering module

We studied three models in the article filtering module (the naïve Bayes classifier, multinomial classifier, and SVM classifier [23]). All of these classifiers require the prior selection of features that represent the data that are to be classified. As mentioned before, there are a large number of term, string, and template features, and thus we use the *χ*^2 ^test to select the most significant ones. The naïve Bayes model for article filtering is defined as follows:

(1) $$\begin{array}{c} R_{NB}(d)=\log\frac{{\mathrm{Pr}}_{NB}(c_{+}|d)}{{\mathrm{Pr}}_{NB}(c_{-}|d)}\\ =\log\frac{\mathrm{Pr}(c_{+})}{\mathrm{Pr}(c_{-})}+\sum_{i=1}^{n}\log\mathrm{Pr}(w_{i}|c_{+})-\sum_{i=1}^{n}\log\mathrm{Pr}(w_{i}|c_{-}) \end{array}$$

Where *d *denotes an article, c_+_/c_- _denotes relevant/irrelevant articles, *w*_*i *_indicates a feature, and *R*_*NB*_(*d*) is the output score indicating the degree of relevance. The multinomial model implies a different distribution:

(2) $$\begin{array}{c} R_{MN}(d)=\log\frac{{\mathrm{Pr}}_{MN}(c_{+}|d)}{{\mathrm{Pr}}_{MN}(c_{-}|d)}\\ =\log\frac{\mathrm{Pr}(c_{+})}{\mathrm{Pr}(c_{-})}+\sum_{i=1}^{n}x_{i}\log\mathrm{Pr}(w_{i}|c_{+})-\sum_{i=1}^{n}x_{i}\log\mathrm{Pr}(w_{i}|c_{-}) \end{array}$$

Where *x*_*i *_denotes the number of times that a feature *w*_*i *_appears in the document *d*. In these two models, we must estimate the probability of each feature appearing in both relevant and irrelevant articles. This can be easily implemented using the equation below:

(3) $$\begin{array}{lll} \mathrm{Pr}(w_{i}|c_{+}) & = & \frac{1+N(w_{i},c_{+})}{V+\sum_{w_{j}}N(w_{j},c_{+})}\\ & = & \frac{1+\sum_{d_{j}\in POS}TF(w_{i},d_{j})}{V+\sum_{w_{k}}\sum_{d_{j}\in POS}TF(w_{k},d_{j})} \end{array}$$

Where *V *is the total number of features, *POS *is the set of relevant documents, and *TF*(*w*_*i*_, *d*_*j*_) is the frequency that the feature *w*_*i *_is observed in the document *d*_*j*_.

These two models are called probabilistic models, because they interpret the data by estimating a probability distribution. As mentioned previously, to analyze the quantitative difference of two distributions, we define the average Kullback Leibler divergence as follows:

(4) $$AKL(p,q)=\frac{1}{2}\sum_{x}\left(q(x)\log\frac{q(x)}{p(x)}+p(x)\log\frac{p(x)}{q(x)}\right)$$

Where *p *and *q *are two distributions over the random variable *x*. If the two distributions are identical, then the *AKL *is 0; otherwise it is positive.

The SVM is a discriminative model, which constructs a hyperplane in the feature space to separate the data into categories. The classification decision is made by calculating the distance of a sample from the hyperplane and in this module; we investigated two types of SVM models. The first is a traditional SVM with a linear kernel and in which each sample is represented as a feature vector. Instead of using TF*IDF, we proposed a new computational scheme to overcome the issue of divergence between the training set and test set, as follows:

(5) $$TF*MLP(w_{i},d_{j})=TF(w_{i},d_{j})*\log\frac{\mathrm{Pr}(w_{i}|c_{+})}{\mathrm{Pr}(w_{i}|c_{-})}$$

Where *TF*(*w*_*i*_, *d*_*j*_) is the frequency of the feature *w*i observed in the document *d*j. The computational scheme performs much better than TF*IDF in the benchmark evaluation. The decision variable in the SVM model is as follows:

(6) $$R_{SVM}(x)=b+\sum_{x_{i}\in SV}y_{i}\alpha_{i}K(x_{i},x)$$

Where *SV *means the support vectors. The kernel function provides an alternative mechanism to represent data in a composite manner in addition to the feature-vector representation. For instance, the *p*-spectrum kernel computes the number of common substrings shared by two input samples [24]:

(7) $$K_{p}(x,y)=<\varphi^{p}(x),\varphi^{p}(y)>=\sum_{u\in \Theta^{p}}\varphi_{u}^{p}(y)*\varphi_{u}^{p}(y)$$

(8) $$\varphi_{u}^{p}(x)=\left|\left\{(v_{1},v_{2})|x=v_{1}uv_{2},u\in \Theta^{p}\right\}\right|$$

Where *x *and *y *are two strings (or documents) defined in the alphabet Θ, and Θ^*p *^indicates all possible substrings of length *p*. In our method, we take *p *= 7, which is about 1.5 times the average length of unigrams. An example of how to construct string features is shown in Table 5. An article here is treated as a string, and no other semantics are considered. This low-level representation reduces the distribution divergence between the training and test data.

**Table 5:** An example for constructing string features

Input and processed documents and condidate string features	Details
Input document	The Three Human Syntrophin Genes Are Expressed in Diverse Tissues, Have Distinct Chromosomal Locations, and Each Bind to Dystrophin and Its Relatives
Processed document	the three human syntrophin genes are expressed in diverse tissues have distinct chromosomal locations and each bind to dystrophin and its relatives
Candidate string features	the thr
	he thre
	e three
	three h
	hree hu
	ree hum
	ee huma
	e human
	...

The length of substring is fixed to 7. The example document only has one sentence (the title of the document of PMID:8576247). A seven-character window moves along the sequential text. All characters are converted to lower case. Only alphabetical letters and the space character are processed. Punctuation is converted to the space character.

### Molecule recognition module

**Figure 4:**
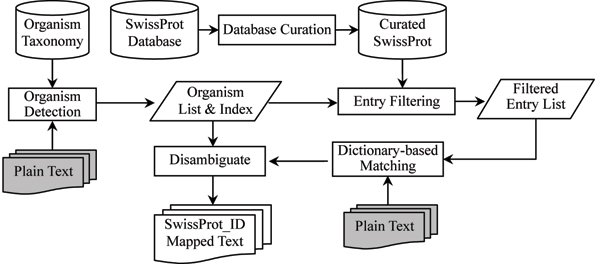
The flowchart of the molecule recognition module. Gray boxes are the input of our molecule recognition module and the figure illustrates the flowchart of the molecule recognition module.

Identifying protein mentions and normalizing them to molecule identifiers is a necessary step toward the extraction of protein interactions. In contrast to traditional named entity recognition tasks, this task requires the submitted protein pairs be mapped to unique SwissProt identifiers rather than presenting the original names in the text. We not only must identify named entities but also we must map them to unique molecule identifiers. As shown in Figure 4, there are four main processes in the molecule recognition module: database curation, organism detection, dictionary-based matching, and removing ambiguity from the mapped names.

After curation, there are in total 230,000 protein identifiers, and more than 1 million terms. Obviously, it is not feasible to use all of the terms during the dictionary-based matching process. Moreover, the same terms, particularly abbreviations, may correspond to many protein identifiers. This is common when the same gene products only differ in organisms, and thus the organism context is crucial to remove such ambiguities. We first detect the organism information in an article, and then this information is used to rule out irrelevant database entries and to remove ambiguities when the terms are mapped to multiple protein identifiers. Our assumption here is that physical interactions described in one paper should occur only within a few organisms. The organism database used here is the NCBI (National Center for Biotechnology Information) taxonomy [25]. Dictionary-based matching is used to detect organisms, and the five most frequent organisms are left such that each sentence can be linked with several detected organisms. To remove the ambiguity from the mapping of the names identified to molecule identifiers, the nearest neighbor principle is used, implying that the organism associated to a recognized name is the organism in the nearest sentence where the name is detected.

Profile-based PPI extraction moduleTo extract experimentally verified physical interactions in practical interaction curation, curators usually collect evidence from multiple sentences. Previous methods to extract protein interactions are all at the sentence level, where each sentence is processed independently, and thus they fail to synthesize information from multiple sentences. Our profile-based method is able to exploit profile features from multiple sources throughout the whole document, and for each candidate interacting protein pair a profile vector is constructed from multiple sentences. In comparison with traditional methods, the profile-based method is more robust for the IPS of BioCreative 2006 and gained the best and the second best results on the SwissProt-only subset and the entire dataset, respectively.

**Figure 5:**
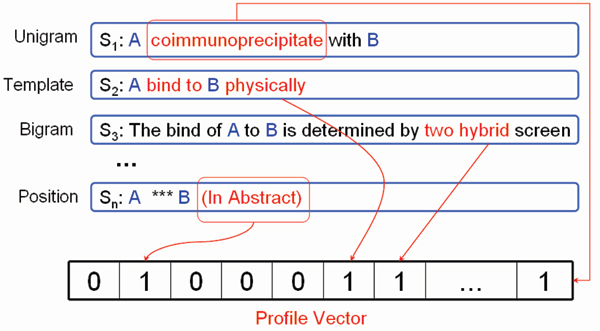
The profile vector in the extraction of interaction protein pairs. The construction of the profile vector for each candidate protein pair is shown in this figure. The term feature (unigram/bigram), template feature, and position feature are used in this process.

Every protein pair coincident in a sentence is viewed as an interaction candidate. For each pair, profile features are calculated from all the sentences in which the pair coincides. The corresponding bit is set to 1 if the feature is found in these sentences (Figure 5). Through such a representation, information from the whole document can be integrated together and a SVM with the linear kernel can be trained on the profile feature vectors. There are three types of profile features.

1. One hundred and sixty-eight unigram/bigram features. One hundred of these features are selected using the *χ*^2 ^test, and 68 are taken manually from the branches of the physical interaction and detection method in the Molecular Interaction (MI) ontology [7].

2. Ninety-one template features. These features are generated in a semi-supervised manner [26] and their form is like 'Protein_1 _* bind to * Protein_2_', where * means that any word can be omitted. Some template examples are listed in Table 6.

**Table 6:** Examples for template features used in the profile-based method

activation of * Protein_1 _* to * Protein_2_
interaction of * Protein_1 _* and * Protein_2_
association of * Protein_1 _* with * Protein_2_
interaction between * Protein_1 _* and * Protein_2_
binding of * Protein_1 _* to * Protein_2_
Protein_1 _* bind * to * Protein_2_
Protein_1 _* activate * Protein_2_
activation of * Protein_1 _* with * Protein_2_
Protein_1 _* coimmunoprecipitate * with * Protein_2_
Protein_1 _* copurify * with * Protein_2_
Protein_1 _* mediate * interaction * Protein_2_
Protein_1 _* form * complex * with * Protein_2_
Protein_1 _* phosphorylate * Protein_2_
Protein_1 _* interact * with * Protein_2_
Protein_1 _* associate * with * Protein_2_
Protein_1 _* in * complex * with * Protein_2_
Protein_1 _* regulate * Protein_2_
regulation of * Protein_1 _* by * Protein_2_
yeast * Protein_1 _* screen * Protein_2_
yeast * strain * Protein_1 _* and * Protein_2_
yeast * Protein_1 _* to * function * Protein_2_
Protein_1 _* lead * to * activation * of * Protein_2_

Template examples are listed in this table. These templates are reproduced from [26]. The complete list of template features is available upon request from the authors. The asterisk in the template features indicates that any word can be skipped.

3. Two position features. One of which is whether the two proteins coincide in the title and the other is whether they coincide in the abstract.

Our method is more robust than the traditional methods because a single description, such as 'Protein_1 _binds to Protein_2_', does not necessarily indicate the existence of a physical interaction. However, if there is other evidence, such as 'The binding of Protein_1 _to Protein_2 _is determined by Y2H', then the interaction is more trustworthy. Clearly, more evidence will strengthen confidence in the interaction. In addition, our algorithm is more robust when the performance of the molecule recognition module is far from satisfactory. For example, in the sentence 'The Y2H experiment proved the interaction between Protein_1 _and Protein_2_, CGA ...', CGA, whichis the sequence of Protein_2_, will be recognized as chromogranin A precursor, and then it will coincide with Protein_1 _and Protein_2_. The previous methods will fail, and although these false pairs are less statistically significant across the whole document, our method is able to resolve the problem by incorporating evidence from multiple sentences.

## Abbreviations

AUC, area under the receiving operator characteristic curve; GN, gene normalization; IAS, interaction article subtask; IMS, interaction method subtask; IPS, interaction pair subtask; MI, Molecular Interaction; PPI, protein-protein interaction; SVM, support vector machine; TF*IDF, term frequency × inverse document frequency weighting.

## Authors' contributions

MH designed the framework and wrote the manuscript. SD implemented the named entity recognition and interaction extraction modules. HW finished the algorithms and methods for the article filtering. XZ made many valuable suggestions and provided much support throughout this work.
